# Self-Perceived Health among School Going Adolescents in Pakistan: Influence of Individual, Parental and Life Style Factors?

**DOI:** 10.5539/gjhs.v5n4p71

**Published:** 2013-04-06

**Authors:** Asad Ali Khan Afridi, Komal Motwani, Saleem Khawaja, Adeel A Khoja, Zafar Fatmi, Iqbal Azam, Muhammad Masood Kadir

**Affiliations:** 1 Aga Khan University, Karachi, Pakistan; 2 Dow University of Health Sciences, Karachi, Pakistan

**Keywords:** adolescents, self-perceived health, life style, Pakistan

## Abstract

**Background:**

Adolescents are at substantial risk of acquiring behaviors which might influence their health status. This study was aimed to assess the proportion of school going adolescents (both males and females) with poor self-perceived health and its associated factors.

**Methodology:**

A cross-sectional study was conducted in three major cities of Pakistan i.e. Karachi, Lahore and Quetta. From each city, six (6) secondary schools were randomly selected (3 public and 3 private). Pre-tested, self-administered questionnaire was distributed to students. Binary logistic regression analysis was conducted to determine independent factors associated with poor self-perceived health.

**Results:**

Approximately 29% adolescents (119/414) reported poor self-perceived health. Individual and parental factors significantly associated with poor self-perceived health were being male (AOR = 1.75, 95% CI: 1.09 – 2.79), living in extended family (AOR = 2.65, 95% CI: 1.66 – 4.22), unskilled employment of father (AOR = 2.17, 95% CI: 1.35 – 3.48), lack of parental-child communication (AOR = 1.74, 95% CI: 1.03 – 2.91) and unfair treatment by parents (AOR = 1.80, 95% CI: 1.09 – 2.96). Life style factors such as use of smokeless tobacco (AOR = 2.14, 95% CI: 1.26 – 3.96) and unhealthy diet (AOR = 3.60, 95% CI: 1.76 – 7.33) were associated with poor self-perceived health.

**Conclusion:**

Better employment opportunities for father, parental counseling and increase awareness for adolescents about healthy diet are recommended to improve adolescent self-perceived health in Pakistan.

## 1. Introduction

There are diverse viewpoints on definition of health comprising of medical, behavioral and socio-environmental perspective. A combination of these constitutes the overlapping models of health (Labonte & Laverack, 2008; Laverack, 2004). World Health Organization declares health as a multidimensional construct unfolded into objective as well as subjective well-being (World Health Organisation. Constitution. Geneva: World Health Organisation, 1946). Consequently, health have been measured on several different dimensions including self-perceived health (Sadana, 2002). Self-perceived health is a reliable and valid indicator for general health and many morbidities and mortality in adults (Barros, Zanchetta, Moura, & Malta, 2009; Idler & Benyamini, 1997). Although widely used among adults, only few studies have been conducted in South Asian population and adolescents using self-perceived scale (Ahmad, Jafar, & Chaturvedi, 2005). Self-perceived scale is also shown to be reliable in adolescents population (Heard, Gorman, & Kapinus, 2008).

Adolescents population is apparently free of disease (Breidablik, Meland, & Lydersen, 2009; Erginoz et al., 2004; Piko, 2000). Nonetheless, they are considered vulnerable for poor self-perceived health as it is a transition period to adulthood (Sawyer et al., 2012). Early adolescence is particularly more vulnerable to poor self-perceived health (Breidablik et al., 2009) as the frequency of subjective health complains upsurges during this period (Haugland, Wold, Stevenson, Aaroe, & Woynarowska, 2001). During this phase health perceptions are also being shaped by continuous changes in bodily, biological, socio-behavioral and mental perspectives (Fatusi & Hindin, 2010; Nollen et al., 2006; Viner et al., 2012). Furthermore, behaviors acquired by adolescents tend to remain with them throughout their life and may result in important health consequences. Approximately 33% of the disease burden and 60% of premature deaths among adults are said to have their roots in adolescent period (Breidablik, Meland, & Lydersen, 2008; Elizabeth Lule, 2006).

Adolescents makes up 22% of the total population of Pakistan (Statistics, 2000-2004). It has fourth largest adolescent population (41 million) in the world, only preceded by United States, China and India (Anthony, 2011). Among adolescent in Pakistan 16% of females are currently married and 33% percent of males and 28% females are enrolled in secondary schools. Early marital age of female adolescents and resulting early pregnancies may result in high infant and maternal mortality.

Unfortunately, there is lack of information on adolescents’ self-perceived health in Pakistan. One previous study conducted among general population in Pakistan also reported self-perceived health among adolescents. This study also lacked information on important parental and lifestyle characteristics of adolescents (Ahmad et al., 2005). Poor health during this critical phase of adolescence may deprive the individual from educational, career and skill building activities that are essential to lead a healthy and economically productive life. The understanding of the variables that shape adolescents’ health perception is essential as it determines the future health scenario in Pakistan. Therefore, our study aimed to assess the proportion of adolescents with poor self-perceived health in schools of three major cities of Pakistan and its association with individual, parental and life style factors.

## 2. Material and Methods

A cross-sectional survey was carried out among 09^th^ and 10^th^ grade students in three capital cities of Pakistan. Four-hundred and thirty-two students were approached in six public and private schools; one each from Karachi, Quetta and Lahore. Amongst students who participated, five were absent on the day of survey and thirteen refused to participate. Thus, we enrolled 414 students which yield response rate of 95%.

Participants were asked to assess their general health with the single-item question ‘In general, are you satisfied with your health?’ This question has been validated in different cultural set-ups (Goodwin & Engstrom, 2002; Subramanian, Subramanyam, Selvaraj, & Kawachi, 2009; Tremblay, Dahinten, & Kohen, 2003). Though recorded on 4 point Likert scale, response options were merged as ‘yes, or no’. For this study, following terms were defined as: 1) Unhealthy dietary intake: < 7 servings of fruits and/or vegetable in last 7 days. 2) Physical inactivity: < 30 minutes of moderate to vigorous activity for less than 4 days in last 7 days. 3) Passive smoking: at least 30 minutes of exposure to second hand smoke most days of the week (at least 5) either at home, public places and/or at school for at least last six months. 4) Current smoking: any number of cigarettes currently smoked either regularly or occasionally. 5) Use of betel nut: any amount of betel nut chewed in last 7 days. 6) Use of smokeless tobacco (SLT): any amount of SLT (oral tobacco, snuff) used in last 7 days. Parents’ education level and occupational groups were also asked. Fathers who were working as professionals, administrators, bankers and teachers were labeled as ‘white collar job’ while those who were working in other categories like: sellers, laborers, and manual workers were labeled as ‘blue collar job’. As very low proportions of mothers were in any occupation, we classified them either in ‘employed’ or ‘house wife’ categories. Family set up was defined as: 1) Nuclear family: family group consisting of only parents and their children and 2) Extended family: family group that includes grandparents, uncles, aunts and cousin besides parents and children. In addition, students were also asked two questions about their parents’ attitude towards them, which may affect students’ self-perception of health: 1) Do you think, your parents communicates well with you and understand your worries and problems? And 2) Do you think, your parents treat you fairly? Both questions were subjective and participants answered in ‘yes’ or ‘no’ categories.

The data was analyzed using Statistical Package for Social Sciences (SPSS) version 19 *(SPSS 19.0; SPSS Inc., Chicago, IL, USA)*. We calculated frequencies and proportions for baseline characteristics of study participants along with parental and lifestyle factors. To determine the difference in risk factors, for poor self-perceived health, by parental and lifestyle factors; chi-square test and odds ratio along with their 95% confidence interval were calculated using binary logistic regression. All those variables that yield p-values of <0.25 at univariate analysis were included in final multivariable logistic regression analysis

### 2.1 Ethical Considerations

The study protocol was approved by the Research Committee of the department of Family Medicine, Aga Khan University, Karachi. Approval was given by the schools’ administration; acceptance from study participants and written informed consent from their parents was taken before start of the study. Pre-tested in a similar area and population group; a structured, self-administered questionnaire was distributed to participants in their classrooms and they were requested to complete it. Before conducting interviews, all study participants and schools were assured about the confidentiality and anonymity and efforts were made to ensure the privacy of the information.

## 3. Results

A total of 414 adolescents were interviewed. The prevalence of poor self-perceived health was 29% (95% C.I.: 24.4, 33.3). The age of the participants ranged between 14 to 17 years with mean age of 14.36 (SD ±1.08) years. More than 70% (297/414) were enrolled in government (matriculate) education system and almost two third (266/414) adolescents lived in a nuclear family set up. Fathers’ of majority of the adolescents (54%) had completed education up to higher secondary school or above whereas only 41% of the mothers had similar level of schooling. About half of the adolescents’ fathers had white collar jobs while huge majority of the mothers (80%) were housewives. Nearly one-third (32%) of the adolescents perceived that their parents had no understanding with them and more than one third (38%) also reported that their parents did not treat them fairly (Table 1).

**Table 1:** Univariate and multivariable analysis for sociodemographic and parental factors associated with poor self-perceived health among adolescents in Pakistan (n=414)

Characteristics	All n (%)	Poor Self perceived health n (%)	p-value^1^	Crude Odds Ratio (95% CI)	Adjusted Odds Ratio (95% CI)
**Sex**			0.032		
Females	191 (46.1)	45 (23.6)		1.0	1.0
Males	223 (53.9)	72 (32.2)		1.61 (1.04-2.49)	1.99 (1.19 – 3.32)
**Type of schooling**			0.694		
O- level	117 (28.3)	32 (27.4)		1.0	1.0
Matric	297 (71.7)	87 (29.3)		1.10 (0.68–1.77)	1.23 (0.69 – 2.16)
**Family**			< 0.001		
Nuclear	266 (64.3)	55 (20.7)		1.0	1.0
Extended	148 (35.7)	64 (43.2)		2.92 (1.88 – 4.54)	2.63 (1.63 – 4.23)
**Fathers’ education**			0.423		
≥ 13 years	225 (54.3)	61 (27.1)		1.0	1.0
<13 years	189 (45.7)	58 (30.7)		1.19 (0.77 – 1.82)	1.05 (0.59 – 1.86)
**Mothers’ education**			0.068		
≥ 13 years	168 (40.6)	40 (23.8)		1.0	1.0
< 13 years	246 (59.4)	79 (32.1)		1.51 (0.97 – 2.36)	0.67 (0.37 – 1.21)
**Fathers’ occupation**			< 0.001		
White collar job	210 (50.7)	43 (20.5)		1.0	1.0
Blue collar job	204 (49.3)	76 (37.3)		2.30 (1.48 – 3.57)	2.08 (1.23 – 3.50)
**Mothers’ occupation**			0.021		
Employed	82 (19.8)	15 (18.3)		1.0	1.0
House-wife	332 (80.2)	104 (31.3)		2.03 (1.11 – 3.73)	1.73 (1.07 – 2.79)
**Parental understanding**			0.020		
Yes	282 (68.1)	71 (25.2)		1.0 1.69 (1.08 – 2.65)	1.0 1.87 (1.08 – 3.22)
No	132 (31.9)	48 (36.4)		1.69 (1.08 – 2.65)	1.87 (1.08 – 3.22)
**Parents fairness with adolescent**			0.012		
Yes	255 (61.6)	62 (24.3)		1.0	1.0
No	159 (38.4)	57 (35.8)		1.74 (1.12 – 2.68)	1.76 ( 1.07 – 2.91)

**p-value^1^:** of chi-squared test

In univariate analysis, sex (p-value = 0.032), type of family (p-value = <0.001), fathers’ occupation (p-value = <0.001), mothers’ occupation (p-value = 0.021), mothers’ education (p-value = 0.068), understanding by parents (p-value = 0.020) and unfair treatment by parents (p-value = 0.012) were found to be significantly associated with poor self-perceived health (Table 1). No significant association was observed for type of schooling (p-value = 0.694), and parents’ education (fathers’ education p-value = 0.423; mothers’ education p-value = 0.068).

In multivariable analysis, odds of having poor self-perceived health were higher among those adolescents who: were males (AOR = 1.99, 95% CI: 1.19 – 3.32), living in extended family (AOR = 2.63, 95% CI: 1.63 – 4.23), whose fathers had unskilled employment (AOR = 2.08, 95% CI: 1.23 – 3.50) and mothers were house wives (AOR = 1.73, 95% CI: 1.07 – 2.79), who reported absence of parental understanding (AOR = 1.87, 95% CI: 1.08 – 3.22) and had unfair treatment by parents (AOR = 1.76, 95% CI: 1.07 – 2.91) were also more likely to perceive poor self-health (Table 1).

The life style characteristics and their association with poor self-perceived health are presented in Table 2. Around a quarter of adolescents reported use of smokeless tobacco, a huge proportion (> 80%) use unhealthy diet, more than one third used betel nuts. Although only about 15% reported active smoking, a greater proportion (>55%) reported passive smoking and approximately 55% reported lack of physical activity in last seven days.

**Table 2:** Univariate and multivariable analysis for life style factors associated with poor self-perceived health among study participants (n=414)

Characteristics	All n (%)	Poor Self perceived health n (%)	p-value^1^	Crude Odds Ratio (95% CI)	Adjusted Odds Ratio (95% CI)
**Use smokeless tobacco**			< 0.001		
No	313 (75.6)	76 (24.3)		1.0	1.0
Yes	101 (24.4)	43 (42.6)		2.31 (1.44 – 3.70)	1.0 2.16 (1.26 – 3.67)
**Use healthy diet**			0.001		
Yes	80 (19.3)	10 (12.5)		1.0	1.0
No	334 (80.7)	109 (32.6)		3.39 (1.68 – 6.83)	3.85 (1.86 – 7.97)
**Betel nuts**			0.025		
No	261 (63.0)	65 (24.9)		1.0	1.0
Yes	153 (37.0)	54 (35.4)		1.64 (1.06 – 2.53)	1.14 (0.66 – 1.97)
**Current smoking**			0.211		
No	355 (85.7)	98 (27.6)		1.0	1.0
Yes	59 (14.3)	21 (35.6)		1.44 (0.81 – 2.59)	1.55 (0.82 – 2.93)
**Passive smoking**			0.315		
Yes	231 (55.8)	71 (30.7)		1.0	1.0
No	183 (44.2)	48 (26.2)		1.24 (0.81 – 1.92)	1.15 (0.72 – 1.86)
**Physical activity**			0.246		
≥ 3 days	189 (45.7)	49 (25.9)		1.0	1.0
0 – 2 days	225 (54.3)	70 (31.1)		1.29 (0.83 – 1.98)	1.23(0.78 – 1.95)

**p-value^1^:** of chi-squared test

In univariate analysis, use of smokeless tobacco (p-value = <0.001), unhealthy diet (p-value = 0.001) and betel nuts (p-value = 0.025) were found to be significantly associated with poor self-perceived health (Table 2). No significant association was observed for current smoking (p-value = 0.211), passive smoking (p-value = 0.315) and physical activity (p-value = 0.246).

In multivariable analysis, however, odds of having poor self-perceived health were higher among those adolescents who used smokeless tobacco (AOR = 2.16, 95% CI: 1.26 – 3.67) and who did not use healthy diet (AOR = 3.85, 95% CI: 1.86 – 7.97) (Table 2).

## 4. Discussion

Our study suggests that poor self-perception of health is more prevalent among males than females and this finding corroborates with previous studies (Jovic-Vranes, Jankovic, Vasic, & Jankovic, 2011; Kestila et al., 2006). Sociodemographic factors such as living in extended family, unskilled employment of fathers, mothers who are housewives, poor parenting (lack of communication) and unfair treatment by parents were found out to be associated factors for poor self-perception. Furthermore, life style factors such as smokeless tobacco usage and unhealthy diet among adolescents are also associated factors with poor self-perceived health. This study identifies the elements that negatively influences one's perception of health, however majority of them are modifiable and may have important public health consequences.

Generally studies report a higher proportion of girls with poor self-perceived health (Piko, 2007; Sweeting & West, 2003). Although this is in contrast to our study findings, it is consistent with finding from study conducted in Serbia (Jovic-Vranes et al., 2011; Kestila et al., 2006; Sweeting & West, 2003). Different studies have reported availability of strong social support to adolescent girls, by elderly female members of their family (Denton & Walters, 1999; Sunder, Ramos, Short, & Rosenthal, 2006) as a major mediating factor for this gender difference in self-perceived health. Also girls commonly enter puberty at younger age (Toppari & Juul, 2010), mature earlier than males (Rogol, Clark, & Roemmich, 2000) and explanation of physiological changes that occur during adolescence, like menstrual cycles (Lee, Chen, Lee, & Kaur, 2006) by elderly females may result in better understanding of their health. Hence, they may be less likely to perceive poor health. This shift in health perceptions may have important economic consequences as males are the primary bread winners in developing countries.

Role of parents and family in upbringing of adolescents cannot be overestimated (Alderfer et al., 2008). It was reported that adolescents who had poor communication with their parents and had unfair treatment from them has poor perception of their health (Richter, Moor, & van Lenthe, 2011; Vilhjalmsson, 1994; Vingilis, Wade, & Adlaf, 1998). Parental economic status and children social well-being has been explored as a mediating factor in a study by Mistry et al. (2002). This is consistent with our study findings, and perhaps indicates the void between perception of care between parents and adolescents. Most likely explanation could be lack of quality time given by parents working in blue collar jobs, to their children, as they are more likely to work long hours and stay away from their children. Conversely, parents in white collar job are likely to spend more quality time with their children.

Different studies have identified family structure as an important factor affecting self-perceived health of adolescents. Generally, adolescents living in families where more social support was present has better self-perceived health (Heard et al., 2008). However, we found that adolescents living in extended family perceive their health poorer as compared to those who live in a nuclear family. It could be due to resource sharing by multiple persons in an extended family system. This may result in a decrease in purchasing power of people on one hand and thinning out of remaining resources on the other hand, limiting them from spending on health related issues.

Furthermore extended family setup may facilitate in acquiring communicable diseases because of overcrowding (Link & Phelan, 1995) which might be translated in to poor health perception.

Adolescents who were living with their parents’ only (nuclear family) might be spending more quality time with them as compared to those who live in an extended family set up; various studies consistently reported that adolescents who are close to their parents had fewer psychological and physical symptoms (Avison & McAlpine, 1992; Heard et al., 2008; Vingilis et al., 1998). On contrary; poor parent-adolescent relationship has been reported as predictor of poor self- perception of health among adolescents (Erginoz et al., 2004; Richter et al., 2011).

Our study results suggest that adolescents whose fathers work in unskilled jobs or mothers are house-wives are more likely to perceive their health as poor. This is in line with findings from other studies (Call et al., 2003; Richter et al., 2011; Sleskova et al., 2006; E. R. Vingilis, Wade, & Seeley, 2002). Employment status is one of the determinants for better socio-economic status of the household (Sleskova et al., 2006). Fathers’ employment in particular may act as a proxy for household income which is very well known to influence health status(E. Vingilis et al., 1998).

The relationship between unhealthy diet and poor self-perceived health has been explored in many studies (Barros et al., 2009; Paulik, Boka, Kertesz, Balogh, & Nagymajtenyi, 2010; Richter et al., 2011) and our results are in agreement with their findings. A study conducted by US Centers for Disease Control and Prevention (CDC), found that less than one quarter of adolescents eat enough fruits and vegetables (Centers for Disease Control and Prevention (CDC). Key strategies for schools to prevent obesity). Results of a Karachi based study also reflects a similar status (Khuwaja, Fatmi, Soomro, & Khuwaja, 2003). This situation warrants the shift from unhealthy diet towards healthier one, by promoting the use of fruits and vegetables.

Use of tobacco in smoked as well as smokeless forms has been documented as a predictor of poor self-perceived health in adults (Ahmad et al., 2005; E. Vingilis et al., 1998). A recent study reported that more than half of adults in Karachi use smokeless tobacco and most of them started it before 15 years of age (N. S. Ali, Khuwaja, T. Ali, & Hameed, 2009). Our study results also found that about one-quarter of adolescents used smokeless tobacco. A little caveat here is not to ignore those adolescents who have unhealthy life styles but perceive their health to be good. Health education, behavior change and rehabilitation services are required to be focused on these individuals as well. These results need immediate attention of policy makers, parents and school administrators for preventing and curbing the ongoing menace which has the potential to jeopardize adolescents’ health.

Findings from this study cannot be generalized and should be interpreted with caution. Selection of schools from major cities limits us to comment on perceptions of adolescents from rural areas. Also, questions on parental understanding and fair treatment had subjective element which may affect objectivity of responses. Self-perceived health could fluctuate substantially depending on how the individual is feeling that day, this could also introduce subjectivity in their responses. Mental health of adolescents was also not looked into; this could be a mediating factor i.e. those adolescents who are depressed have negative self-perceived health and negative views of their relationships with their parents. However, it is one of the few studies addressing adolescents’ self-perceived health from a developing country like Pakistan; which included schools both from public and private sector to get a wider representation of population.

## 5. Conclusion

In conclusion, this study highlights that a substantial proportion of our adolescents perceive their health as poor, which can potentially affect their health and wellbeing in future. Focusing attention on health promoting behaviors like use of fruits and vegetables and increase physical activity along with emphasis on strengthening relationship with parents may result in a positive perception of their health by adolescents. In this regard, school-based counseling and mass media campaigns may play a pivotal role.
